# First person – Flavie Ader

**DOI:** 10.1242/bio.059644

**Published:** 2022-10-03

## Abstract

First Person is a series of interviews with the first authors of a selection of papers published in Biology Open, helping researchers promote themselves alongside their papers. Flavie Ader is first author on ‘ Drosophila CRISPR/Cas9 mutants as tools to analyze cardiac filamin function and pathogenicity of human FLNC variants’, published in BiO. Flavie is an associate professor and hospital practitioner in the lab of Professor Philippe Charron at Pitié Salpétrière APHP University Hospital, Paris, France, investigating genetic and physiopathology of cardiomyopathies.


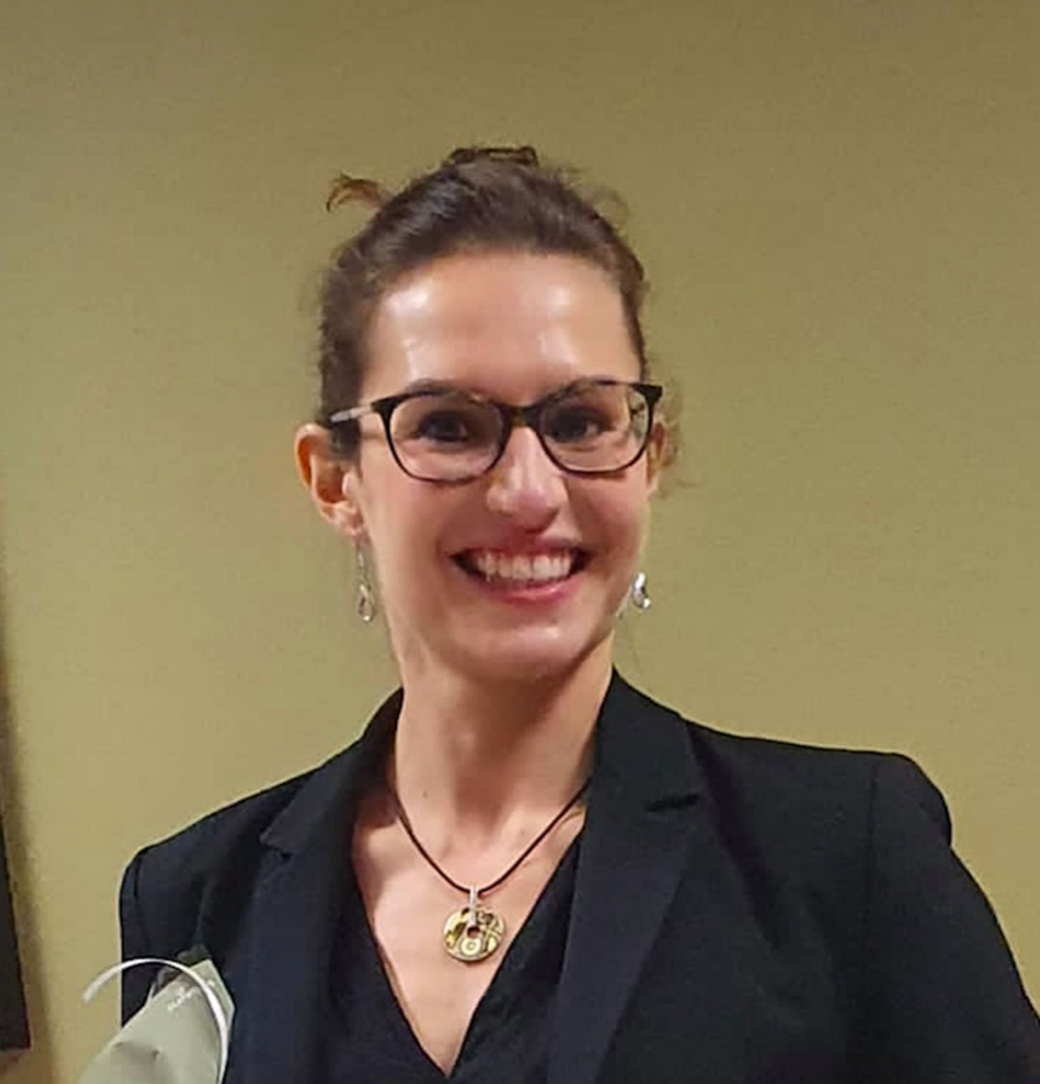


**Flavie Ader**

**Describe your scientific journey and your current research focus**

I studied pharmacy at university 15 years ago and I specialize in molecular biology. After 2 years in a laboratory dedicated to the molecular diagnosis of cardiomyopathy at Pitié Salpétrière University Hospital (in the functional unit of Dr Pascale Richard), thanks to the close links with the research team, I started a PhD in order to improve my knowledge of the genetic of cardiomyopathies and also the technics.

**Who or what inspired you to become a scientist?**

I'm curious and I always wanted to know how things work at the microscopic level. The genetic field responds to this criterion as molecular changes could impact system and morphology.

**How would you explain the main finding of your paper?**

It's sometimes difficult to confirm which part/region of a protein interacts with other proteins or which part of a protein is responsible for a specific function. In this work, we have evidence that the terminal part of cheerio is not mandatory for heart function.

**What are the potential implications of this finding for your field of research?**

Well-defined protein region function is essential before using targeted therapeutics. Targeted therapeutics, gene or mutation dependent, are in development, thus this work contributes to defining which parts of proteins are essential or could be deleted.

**Figure:**
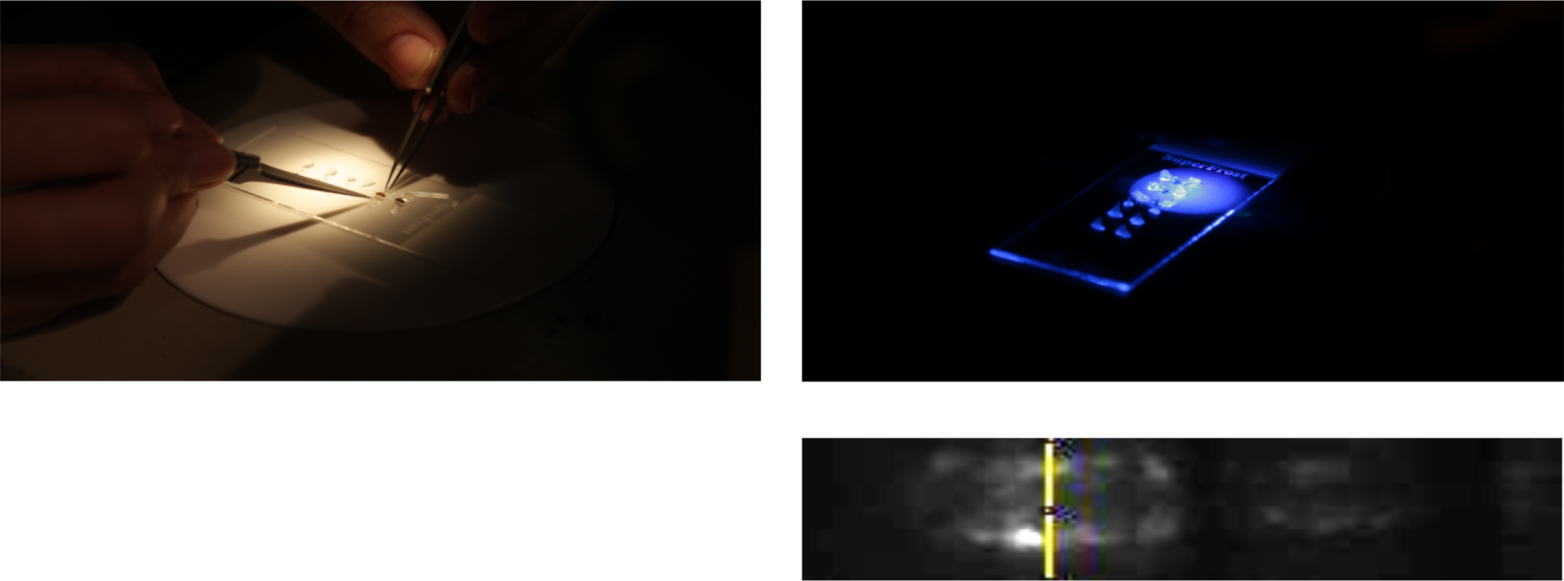
**The modified flies are asleep, placed on a glass slide with tongs.** They expressed a cardiac fluorescent protein and after recording with fluorescent stereo-microscope, their heartbeats can be analyzed.

**Which part of this research project was the most rewarding?**

The collaboration with a team with extensive experience in non-mammalian models, and the tools they used was an opening for me.

**What do you enjoy most about being an early-career researcher?**

I love the collaboration with people with complementary competencies and meeting people with really interesting and various backgrounds. I've met passionate people.

**What piece of advice would you give to the next generation of researchers?**

Keep positive, and rigorous. A lot of amazing tools are developing and will improve knowledge and open therapeutic options for diseases. But we have to keep in mind to be rigorous for some technologies of which we have less background knowledge.

**What's next for you?**

I'll take an academic position in a university hospital as well as at the university as an associate professor and hospital practitioner in the field of cardiogenetics.
